# Potential and Current Distributions Calculated Across a Quantum Hall Effect Sample at Low and High Currents

**DOI:** 10.6028/jres.100.040

**Published:** 1995

**Authors:** M. E. Cage, C. F. Lavine

**Affiliations:** National Institute of Standards and Technology, Gaithersburg, MD 20899-0001

**Keywords:** breakdown of dissipationless state, charge distributions, current distributions, logarithmic charge-redistribution potential, parabolic confining potential, potential distributions, quantum Hall effect, two-dimensional electron gas

## Abstract

The potential and current distributions are calculated across the width of a quantum Hall effect sample for applied currents between 0 μA and 225 μA. For the first time, both a confining potential and a current-induced charge-redistribution potential are used. The confining potential has a parabolic shape, and the charge-redistribution potential is logarithmic. The solution for the sum of the two types of potentials is unique at each current, with no free parameters. For example, the charge-depletion width of the confining potential is determined from a localization experiment by Choi, Tsui, and Alavi, and the spatial extent of the conducting two-dimensional electron gas across the sample width is obtained from the maximum electric field deduced from a high-current breakdown experiment by Cage and Lavine, and from the quantum Hall voltage. The spatial extent has realistic cut-off values at the sample sides; e.g., no current flows within 55 magnetic lengths of the sides for currents less than 215 μA. The calculated potential distributions are in excellent agreement with contactless electro-optic effect laser beam measurements of Fontein et al.

## 1. Introduction

The potential and current distributions within quantum Hall samples are important aspects of the integer quantum Hall effect [1–3]. In this effect the Hall resistance, *R*_H_, of the *i*th plateau of a fully quantized two-dimensional electron gas (2DEG) has the value *R*_H_(*i*)=*h*/(*e*^2^*i*), where *h* is the Planck constant, *e* is the elementary charge, and *i* is an integer. Early attempts to measure potential distributions across samples [4–7] used electrical contacts to the two-dimensional gas that were placed within the sample interior. The potentials were found to vary throughout the entire sample. There was concern, however, that the electrical contacts themselves significantly altered the potential distributions. Fontein et al. [8] have made contactless measurements of potential distributions using a laser beam and the electro-optic Pockels effect. They observed major fractions of the quantum Hall voltage occurring near the sides of the sample, but also significant contributions within the interior. Valid predictions of the potential distribution across quantum Hall samples should agree with their results.

In this paper we calculate the potential distributions across the sample for applied currents *I*_SD_ between 0 μA and 225 μA by: (a) assuming a parabolic confining potential for the charge carriers and using parameters of the parabola obtained experimentally by Choi, Tsui, and Alavi [9]; (b) assuming an applied current-induced logarithmic charge-redistribution potential for the charge carriers that is similar to that of Balaban, Meirav, and Shtrikman [10], but with very different cut-off values for the spatial extent of the potential; (c) assuming that the width of the conducting region varies with applied current because a Lorentz force deflects the conducting electrons slightly towards one side of the sample; (d) using the maximum electric field deduced by Cage and Lavine [11] from a breakdown experiment at high currents to determine the cut-off value for the spatial extent on one side of the sample; and (e) using the quantum Hall voltage to determine the cut-off value of the spatial extent on the other side of the sample. The calculated potential distributions are in excellent agreement with the measurements of Fontein et al. [8].

## 2. Coordinate System

**Fig. 1:**
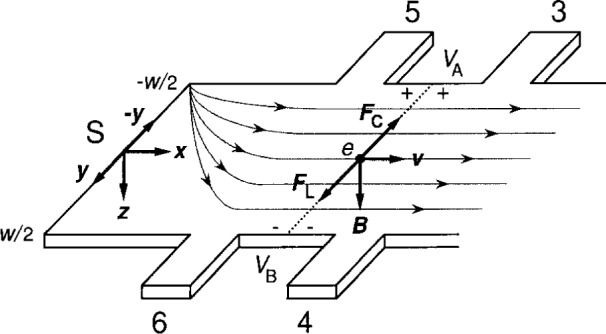
The coordinate system used in this paper. Only part of the sample is shown. The origin is located at the source S, halfway across the sample width *w*. The dotted curves indicate the electron flow pattern for this magnetic field direction. *F*_L_ is the Lorentz force on the conducting electrons and *F*_C_ is the Coulomb force. *B* is the magnetic field, *v* is the electron velocity, and *V*_A_ and *V*_B_ are the potentials on either side of the sample.

The coordinate system is shown in Fig. 1. For convenience in writing the equations, the origin is located at the source S and is halfway across the sample width *w.* The sample labeling is chosen to be consistent with previous work [11]. Potential probes 1 and 2, and the drain D, are not shown. The positive *x* axis points along the sample in the general direction of the externally applied current *I*_SD_. The positive *y* axis is chosen as indicated. Therefore the positive *z* axis points downward for a right-handed coordinate system. The magnetic field *B* also points downward, simply to be consistent with results from the breakdown experiment [11] that will be used in Secs. 4 and 5.

Note that the conducting charges are *electrons* with charge *q* = − *e*. This is taken into account throughout the paper; it is necessary to do so because the signs of both the confining potential and the charge-redistribution potential depend on the sign of the charge carriers.

The Lorentz force ***F***_L_ = *e****v*** × ***B*** is in the positive *y* direction. This force deflects the electrons slightly to the right until it is matched by the Coulomb repulsive force ***F***_C_ = − *e****E*** [12]. A charge-redistribution of the 2DEG results from this deflection. Also because of the Lorentz and Coulomb forces, the electrons enter the corner of the source at *y* = − *w*/2 for this magnetic field direction and exit at the corner +*w*/2 of the drain—in agreement with the experiment of Klass et al. [13]. We assume that the electrons spread out across the sample interior in agreement with the experiment of Fontein et al. [8]. Potential probes 4 and 6 are near the potential of the source. Probes 3 and 5 are near the potential of the drain, and have a positive potential relative to the source for these current and magnetic field directions. The chemical potential *ϕ*_A_ = *V*_A_ is therefore positive relative to the chemical potential *ϕ*_B_ = *V*_B_ on the opposite side of the sample.

## 3. Confining Potential

We begin the calculations with a confining potential to prevent the 2DEG from spilling out the sides of samples. Choi, Tsui, and Alavi [9] performed an experiment on mesa-etched GaAs/AlGaAs heterostructure samples in zero magnetic field. They then used one-dimensional localization theory to deduce the charge-depletion widths, ***Δ***, of the confining potentials, and found that ***Δ*** was (0.5 ± 0.2) μm for a 2DEG of surface number density *n*_s_ = *i*(*eB*/*h*) = 1.5 × 10^11^/cm^2^. We will use their results to define the depletion width of the confining potential for a mesa-etched sample.

### 3.1 Charge-Depletion Region

**Fig. 2:**
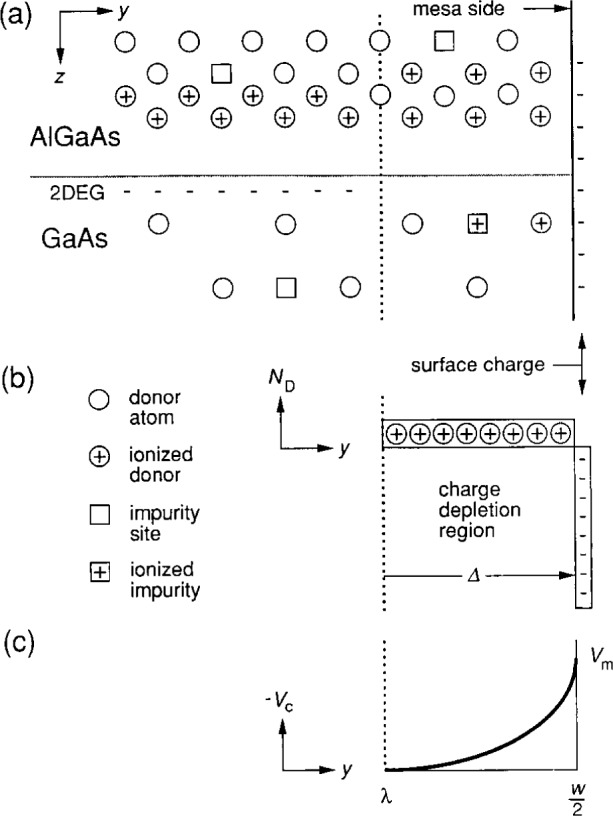
(a) Schematic diagram of the GaAs/AlGaAs interface region near one side of the mesa. See Sec. 3.1 for further explanation. (b) The ionized donor charge-depletion density distribution *N*_D_. (c) The confining potential *V*_c_ for negatively charged surface states.

Figure 2(a) shows a schematic of the charge distribution in the GaAs/AlGaAs interface region near one side of the mesa when there is no applied magnetic field. The GaAs layer of our sample [11] has a residual donor density of about 1 × 10^14^/cm^3^, while the donor concentration in the AlGaAs layer is about 1 × 10^18^/cm^3^ and *n*_s_ = 5.94 × 10^11^/cm^2^. There is an ionized donor atom in the AlGaAs layer for every electron in the 2DEG but, unlike Choi, Tsui, and Alavi [9], we assume the ionized donor atoms are distributed over a volume rather than in a surface sheet with density *n*_s_. The confining potential is generated from electron surface charges on the side of the mesa, as indicated in the figure. There is an ionized donor atom or ionized impurity site in the charge-depletion region for every surface charge.

We assume a homogeneous charge-depletion region in Fig. 2(b). The depletion width ***Δ*** for a homogeneous three-dimensional material is [14]

$$\Delta ={\left(2\varepsilon_{s}V_{m}/eN_{D}\right)}^{1/2},$$ (1)

where ***ε***_s_ = ***κε***_0_ is the dielectric permittivity of the semiconductor, ***κ*** is the dielectric constant (***κ*** = 13.1 for GaAs), ***ε***_0_ is the permittivity of vacuum, *V*_m_ is the value of the confining potential at ±*w*/2 and *N*_D_ is the average density of ionized donors and impurity sites in the charge-depletion region. We selected the value of the charge-depletion width to be ***Δ*** = 0.5 μm [15]. This value is consistent with the results of Choi, Tsui, and Alavi [9]. We chose the value of *V*_m_ to be one-half the 1.50 V separation between the valence and conduction bands of GaAs at 1 K [14], or *V*_m_ = 0.75 V. The value of the average charge-depletion density from Eq. (1) is thus *N*_D_ = 4.3 × 10^15^/cm^3^, which seems quite reasonable.

### 3.2 Confining Potential Equation

A homogeneous charge-depletion region results in a parabolic confining potential *V*_c_, with the origin at *y = λ = w*/2−***Δ***, as indicated schematically in Fig. 2(c). The confining potential is *negative* because the charges on the side of the mesa are electrons.

The equations for the confining potential *V*_c_ and its electric field *E*_c_ = − ***∇****V*_c_ are

$$V_{c}(y)=-a{\left(y-\lambda \right)}^{2}\;\text{and}\;E_{c}(y)=2a(y-\lambda )$$ (2a)

$$\begin{array}{c} \text{for}\;\lambda \leq y\leq \frac{w}{2},\\ V_{c}(y)=0\;\text{and}\;E_{c}(y)=0 \end{array}$$ (2b)

$$\begin{array}{c} \text{for}-\lambda <y<\lambda ,\\ V_{c}(y)=-a{\left(y+\lambda \right)}^{2}\;\text{and}\;E_{c}(y)=2a(y+\lambda )\\ \text{for}-\frac{w}{2}\leq -y\leq -\lambda , \end{array}$$ (2c)

where *a* = *V*_m_/***Δ***^2^ = 3.0 × 10^12^ *V*/m^2^ for ***Δ*** = 0.5 μm and *V*_m_ = 0.75 V, and

$$\lambda =\frac{w}{2}-\Delta .$$ (3)

### 3.3 Confining Potential at *I*_SD_ = 0 *μ*A

Given the values of ***Δ*** and *V*_m_, there is a surprising amount that can be deduced about the electron states of the confining potential when the magnetic field is adjusted to be at the center of the *i* = 2 quantum Hall plateau and *I*_SD_ = 0 μA. Since there is no applied current, and therefore no Hall voltage, the Fermi energy ***ε***_F_ is constant across the sample width and is located halfway between Landau levels. Under these conditions, states of the lowest (*N* = 0) Landau level are occupied up to the Fermi energy ***ε***_F_ = *ħω*_c_/2, no states are occupied in the second (*N* = 1) Landau level, *ω*_c_ = eB/m* is the cyclotron angular frequency, m* is the reduced mass of the electron (0.068 times the free electron mass in GaAs), and *ħ* ≡ *h*/2*π*. References [11,16–18] describe how these states can be defined in the Landau gauge.

**Fig. 3:**
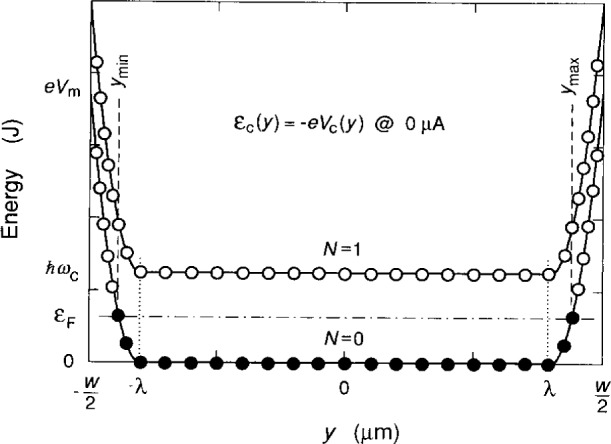
Schematic drawing of the energy of the confining potential *V*_c_ across the sample when *I*_SD_ = 0 μA. Values of the charge-depletion width *Δ* and the Landau energy level spacing *ħω*_c_ are greatly exaggerated. The occupied/unoccupied states of the first two Landau levels are shown as solid/open circles. The occupied (filled) states lie between the locations *y*_max_ = − *y*_min_.

Figure 3 shows a schematic drawing of the energy of the confining potential for *I*_SD_ = 0 μA, with greatly exaggerated values of ***Δ*** and *ħω*_c_, and only a small fraction of allowed states. The occupied/unoccupied states are indicated as solid/open circles, and the occupied (filled) states lie between *y*_max_ and *y*_min_ = − *y*_max_. In the presence of the magnetic field, electrons of the 2DEG occupy Landau level states that penetrate into the charge-depletion regions near the mesa edge, and current circulates around the sample periphery. Under these conditions

$$\begin{array}{c} \varepsilon_{c}(y_{\max})=\frac{\hbar \omega_{c}}{2}=-eV_{c}(y_{\max})=ea{\left(y_{\max}-\lambda \right)}^{2}\\ =e\frac{V_{m}}{\Delta^{2}}{\left(y_{\max}-\lambda \right)}^{2}, \end{array}$$ (4)

where *λ* = *w*/2 − **Δ**.

The occupied states of the right-hand side (rhs) confining potential generate a total current *I*_c_ (rhs) that is

$$\begin{array}{c} I_{c}(\text{rhs})\int_{\lambda }^{y_{\max}}J_{c}(y)dy=\int_{\lambda }^{y_{\max}}\sigma_{xy}E_{c}(y)dy=\\ -\frac{1}{R_{H}}[V_{c}(y_{\max})-V_{c}(\lambda )],\;=-\frac{V_{c}(y_{\max})}{R_{H}}, \end{array}$$ (5)

where *J*_c_(*y*) is the current density, *σ_xy_* is the off-diagonal conductivity tensor component, *V*_c_(*y*_max_) = − *a*(*y*_max_ − *λ*)^2^, and *V*_c_(*λ*) = 0. In the absence of significant dissipative scattering on the quantum Hall plateau, *σ*_xy_ = 1/*R*_H_ [12]. Similarly,

$$\begin{array}{c} I_{c}(\text{lhs})=\int_{y_{\min}}^{-\lambda }J_{c}dy=\int_{y_{\min}}^{-\lambda }\sigma_{xy}E_{c}(y)dy=\\ -\frac{1}{R_{H}}[V_{c}(-\lambda )-V_{c}(y_{\min})],\;=-\frac{V_{c}(y_{\min})}{R_{H}}, \end{array}$$ (6)

where *V*_c_(*y*_min_) = − *a*(*y*_min_ +*λ*)^2^.

It follows from Eqs. (2) to (6) for the 12 906.4 Ω, *i* = 2 plateau at 12.3 T, for the 400 μm wide sample of Ref. [11], and for *I*_SD_ = 0 μA that

$$I_{c}(\text{rhs})=\frac{\hbar \omega_{c}}{2eR_{H}}=\frac{ie^{2}B}{4\pi m*}=0.81\;\mu A=-I_{c}(\text{lhs}),$$ (7)

$$y_{\max}=-y_{\min}=199.559\;\mu m,$$ (8)

and

$$\frac{w}{2}-y_{\max}=0.441\;\mu m.$$ (9)

Thus, a rather large 0.81 μA current circulates around the sample at 12.3 T when *I*_SD_ = 0 μA, ***Δ*** = 0.5 μm and *V*_m_ = 0.75 V. The maximum extent of this current is 60 times farther from the sides of the sample than that produced by skipping orbits bouncing off of a hard wall with a cyclotron radius or magnet length *l_B_* = (*ħ*/*eB*)^1/2^ of 7.3 nm.

## 4. Charge-Redistribution Potential

Section 2 noted that the Lorentz force exerted on the conducting electrons causes deviations − *e*δ*σ*(*y*) from the average surface charge density − *eσ*_ave_ = − *en*_s_ = − *ie*^2^*B*/*h* of the 2DEG charge-redistribution across the sample width. The resulting charge-redistribution potential, *V*_r_(*y*), arising from applied currents would be a linear function of *y* if the mobile electrons occupied a three-dimensional volume. They occupy a two-dimensional sheet, however, and MacDonald, Rice, and Brinkman [19] expressed this charge-redistribution self-consistently in terms of a charge-redistribution potential as

$$V_{r}(y)=-\frac{e}{2\pi \kappa \varepsilon_{0}}\int_{-w/2}^{w/2}\delta \sigma (y^{\prime })\ln\left[\frac{2}{w}\left|y^{\prime }-y\right|\right]dy^{\prime },$$ (10)

where

$$\delta \sigma (y)=\frac{im*}{hB}\frac{d^{2}}{dy^{2}}V_{r}(y)=\frac{ie}{h\omega_{c}}\frac{d^{2}}{dy^{2}}V_{r}(y),$$ (11)

as shown in Appendix A. Riess [20] extended this potential to a 2DEG with finite thickness. Thouless [21] then found an analytic logarithmic approximation of this potential far from the sample sides, and Beenakker and van Houten [22] then approximated the near-edge behavior by introducing a cut-off at a distance *ξ* from the sample side, and a linear extrapolation for

$$V_{r}(y)=-\xi \int_{-w/2}^{w/2}\frac{d^{2}}{d{y^{\prime }}^{2}}V_{r}(y^{\prime })\ln\left[\frac{2}{w}\left|y^{\prime }-y\right|\right]dy^{\prime }$$ (12)

from |*y*| = *w*/2 − *ξ* to |*y*| = *w*/2. The characteristic length *ξ* is $\xi =il_{B}^{2}/\pi a*=ie^{2}/(2\pi \kappa \varepsilon_{0}h\omega_{c})$, where *l_B_* = (*ħ*/*eB*)^1/2^ is the magnetic length and a* = 4*πκε*_0_ *ħ*^2^/m*e^2^ is the effective Bohr radius in SI units. Our values of ξ, *l*_B_, and a* for the *i* = 2 plateau at 12.3 T are 3.3 nm, 7.3 nm, and 10.2 nm, respectively.

Balaban, Meirav, and Shtrikman [10] used a nonlinear (quadratic) extrapolation near the sample sides and obtained the charge-redistribution potential

$$V_{r}(y)=-\frac{I_{\text{SD}}R_{H}}{2}{\left[\ln\frac{w}{\delta }+\frac{\xi +\delta }{2\xi }\right]}^{-1}\ln\left|\frac{y+w/2}{y-w/2}\right|$$ (13)

for |*y*| < *w*/2 − *δ*, where *δ* = *l_B_* for the *i* = 2 plateau, and *δ* is not the differential *δ* of Eq. (10). They successfully used this potential to describe the sample-width dependence for breakdown at small currents, but could not account for the larger breakdown currents observed in other experiments [11, 23–28]. Their geometry factor is

$${\left[\ln\frac{w}{\delta }+\frac{\xi +\delta }{2\xi }\right]}^{-1}=0.08$$ (14)

for our values of *ξ* and *δ* at *w* = 400 μm.

### 4.1 Charge-Redistribution Potential Equation

The charge-redistribution potential described by Eq. (13) was calculated for an infinite square-well confining potential, and must be modified for use with a more realistic confining potential. To do this correctly would require a numerical solution of Eq. (12), with the confining potential included, as is discussed in Appendix A. We approximated this numerical solution (and then tested the approximation) by using the form of the potential in Eq. (13) but introducing two parameters, *y*_min_ and *y*_max_, that alter the charge-redistribution potential due to the presence of the quadratic confining potential.

It was necessary to do this because the potential distribution of Eq. (13), with a cut-off distance *δ* = *l_B_*, gave the correct quantum Hall voltage *V*_H_ = *R*_H_*I*_SD_ across the sample, but the electric field *E*_r_ = − **∇***V*_r_ did not increase quickly enough for increasing current to satisfy the *I*_SD_ = 0 μA conditions of Sec. 3.3 and then reach the electric field values necessary for quasi-elastic inter-Landau level scattering (QUILLS) transitions [11, 16–18,25–28] at high currents.

We use the same form for the charge-redistribution potential as Balaban et al. [10], but with a different geometrical factor and very different cut-off values, *y*_min_ and *y*_max_, which vary with applied current. Our charge-redistribution potential is

$$V_{r}(y)=-\frac{I_{r}R_{H}}{2}{\left[\ln\frac{y_{\max}+w/2}{w/2-y_{\max}}\right]}^{-1}\ln\left|\frac{y+w/2}{y-w/2}\right|,$$ (15)

$$\text{for}\;-\frac{w}{2}<y_{\min}\leq y\leq y_{\max}<\frac{w}{2}$$

$$\text{where}\;I_{r}=I_{\text{SD}}-I_{c}(\text{rhs})-I_{c}(\text{lhs}).$$ (16)

*I*_c_(rhs) and *I*_c_(lhs) are defined by Eqs. (5) and (6), and the geometry factor *G* in Eq. (15) is

$$G(w,y_{\max})={\left[\ln\frac{y_{\max}+w/2}{w/2-y_{\max}}\right]}^{-1}.$$ (17)

We assume *G* is current-independent, and assign the value

G=0.147 (18)

to Eq. (17) by using the value of *y*_max_ = 199.559 μm found in Sec. 3.3 for *I*_SD_ = 0 μA and *w* = 400 μm. Our value of *G* is thus somewhat larger than the value *G* = 0.08 that would be used by Balaban et al. [10]. The cut-off values

$$\delta_{max}=w/2-y_{\max}\;\text{and}\;\delta_{\min}=w/2+y_{\min}$$ (19)

will be determined in Sec. 5. Appendix B discusses the agreement between our Eq. (15) and the self-consistent Eqs. (10) and (11).

The electric field *E*_r_ = − **∇***V*_r_ due to redistribution of the 2DEG with applied current is

$$E_{r}(y)=\frac{I_{r}R_{H}}{2}G\frac{w}{\left[{\left(w/2\right)}^{2}-y^{2}\right]}.$$ (20)

We now have nearly all the information necessary to determine the potential and current distributions.

## 5. Calculations

**Fig. 4:**
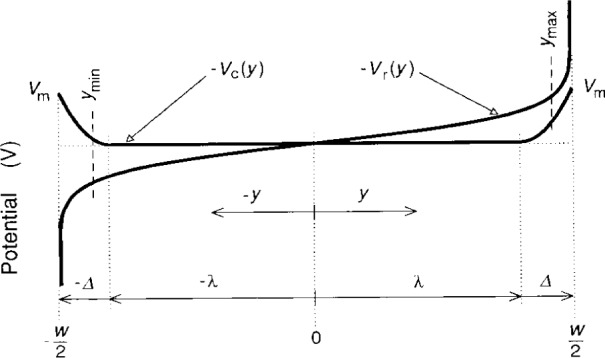
The confining potential − *V*_c_(*y*) and the charge-redistribution potential − *V*_r_(*y*) across the sample for greatly exaggerated values of ***Δ***, *δ_max_ = w*/2 − *y*_max_, and *δ*_min_ = *w*/2 + *y*_min_.

Figure 4 shows the confining potential − *V*_c_(*y*) and the charge-redistribution potential − *V*_r_(*y*) across the sample for greatly exaggerated values of ***Δ***, *δ*_max_, and *δ*_min_, and for an arbitrary value of *I*_r_, where *I*_r_ is defined by Eq. (16). *V*_r_ becomes infinite at ±*w*/2, but that is of no concern because it is only the *occupied states* which contribute to the Hall voltage, and those states occur only between *y*_max_ and *y*_min_. The potentials are therefore finite and well-behaved in the region of interest.

### 5.1 Total Potential

Of course the electrical transport properties depend on the *total* potential *V*_t_(*y*), but we can unambiguously separate *V*_t_(*y*) into the confining and charge-redistribution potential components

$$V_{t}(y)=V_{c}(y)+V_{r}(y).$$ (21)

We have uniquely defined the potentials *V*_c_(*y*) and *V*_r_(*y*) in Eqs. (2) and (3) of Sec. 3.2 and Eqs. (15) to (18) in Sec. 4.1, plus Eqs. (5) and (6) in Sec. 3.3. The current-independent parameters for the confining potential and the charge-redistribution potential are: ***Δ*** = 0.5 μm, *V*_m_ = 0.75 V, and *G* = 0.147. For a given sample we know the applied current *I*_SD_ and the sample width *w*, but there are still two free parameters: *y*_max_ and *y*_min_.

Ordinarily, it would not be possible to uniquely determine the values of *y*_max_ and *y*_min_ since the only other piece of information is that the quantum Hall voltage *V*_H_ is

$$V_{H}=R_{H}I_{\text{SD}}=V_{t}(y_{\min})-V_{t}(y_{\max}),$$ (22)

and there is a range of values for *y*_max_ that satisfies this equation. It *is* possible, however, to determine the value of *y*_max_ for a particular type of experiment, and we believe that the results are representative of most other experiments since our calculations agree with the experimental data of Fontein et al. [8]. We first note that *E* (*y*) = − ***∇****V*(*y*). Therefore

$$E_{t}(y_{\max})=E_{c}(y_{\max})+E_{r}(y_{\max}).$$ (23)

In an experiment described in Ref. [11] we measured the quantized longitudinal voltage drops along a GaAs/AlGaAs sample between potential probes 4 and 6 of Fig. 1 at high currents, and deduced the maximum electric field *E*_max_ from a quasi-elastic inter-Landau level scattering model. The results were

$$E_{\max}=1.1\times 10^{6}\text{V/m}\;@\;I_{\text{SD}}=215\;\mu A$$ (24a)

and

$$E_{\max}=4.2\times 10^{6}V/m\;@\;I_{\text{SD}}=225\mu A.$$ (24b)

The value *E*_max_ = 1.1×10^6^ V/m at *I*_SD_ = 215 μA was just sufficient to excite the lowest, *M* = 1, QUILLS transitions [11,25–28]. It is clear from Fig. 4 that *E*_max_ will occur at *y*_max_, so

$$E_{t}(y_{\max})=E_{\max}.$$ (25)

We can therefore use Eqs. (23) and (24) to determine *y*_max_, and then Eq. (22) to obtain *y*_min_ for the sample of Ref. [11]. Note that changing the values of *y*_max_ and *y*_min_ also alters the values of *I*_c_(rhs), *I*_c_(lhs), and thereby the value of *I*_r_ in Eqs. (5), (6), and (16). Thus there are *no free parameters*, and one can obtain unique solutions to the total potential and other transport properties.

### 5.2 Results

Relevant values for the solution at *I*_SD_ = 0 μA are shown in Table 1. Most were calculated in Sec. 3.3; the remainder were found from Eqs. (2), (3), and (15) to (22). Note that *y*_max_ and *y*_min_ are predicted to be about 60 magnetic lengths from the sides of the sample.

We calculate the values shown in Table 1 at *I*_SD_ = 215 μA by increasing the value of *y*_max_ until *E*_t_(*y*_max_) = 1.1×10^6^ V/m, adjusting the value of *y*_min_ to obtain the correct Hall voltage, and remembering that changing the values of *y*_max_ and *y*_min_ also changes the values of *I*_c_(rhs), *I*_c_(lhs), and *I*_r_. The solution is unique, with no free parameters. The same procedure is done at *I*_SD_ = 225 μA, except that the value of *y*_max_ is increased until *E*_t_(*y*_max_) = 4.2×10^6^ V/m. Note in Table 1 that *y*_max_ is still about 13 magnetic lengths away from the side of the sample at *I*_SD_ = 225 μA.

We also calculate the relevant quantities at *I*_SD_ = 25 μA, which is a current often used in precision quantized Hall resistance measurements. In this case, however, we do not know the value of *E*_t_(*y*_max_), so we use a linear interpolation of the value of *y*_max_ between its values for *I*_SD_ = 0 μA and 215 μA. The quantities shown in Table 1 for *I*_SD_ = 25 μA are relatively insensitive to this choice for *y*_max_.

### 5.3 Plots

**Fig. 5:**
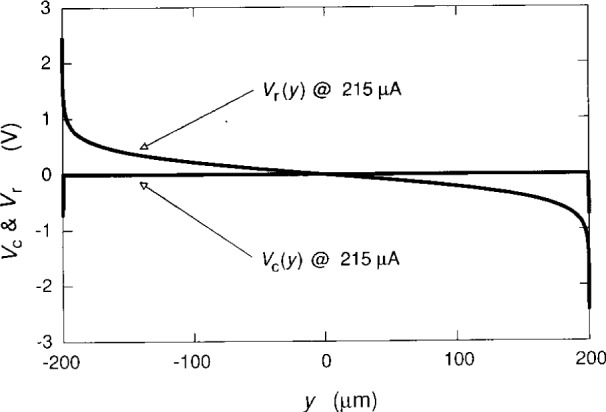
*V*_c_(*y*) and *V*_r_(*y*) plotted between ±0.99999 *w*/2 for the parameters used in Table 1 at *I*_SD_ = 215 μA. The parameters common to all plots in Figs. 5–11 are *i* = 2 (12 906.4 Ω), *B* = 12.3 T, *w* = 400 μm, ***κ*** = 13.1, ***Δ*** = 0.5 μm, *V*_m_ = 0.75 V, and *G* = 0.147.

**Fig. 6:**
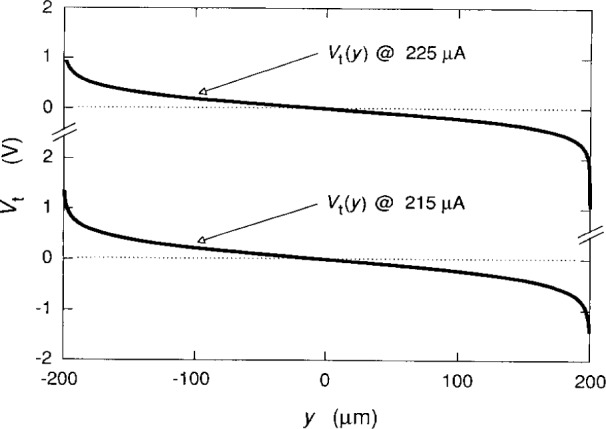
*V*_t_(*y*) plotted between *y*_max_ and *y*_min_, using the parameters in Table 1 for *I*_SD_ = 215 μA and 225 μA. The values of *y*_max_ and *y*_min_ are 199.599 μm and −199.515 μm, and 199.901 μm and −198.044 μm for *I*_SD_ = 215 μA and 225 μA, respectively.

We now plot the potentials, using Eqs. (2), (3), (15) to (19), and (21). Figure 5 shows *V*_c_(*y*) and *V*_r_(*y*) for the parameters used in Table 1 at *I*_SD_ = 215 μA, except that the plot is between ±0.99999 *w*/2 (±199.998 μm) rather than *y*_max_ and *y*_min_ in order to show the sharpness of the confining potential and the extent of the charge-redistribution potential at these extreme values of *y*. Figure 6 shows *V*_t_(*y*) plotted between *y*_max_ and *y*_min_ using the parameters in Table 1 at *I*_SD_ = 215 μA and 225 μA. Other than moving farther to the right, the total potential does not significantly change shape with increasing current.

**Fig. 7:**
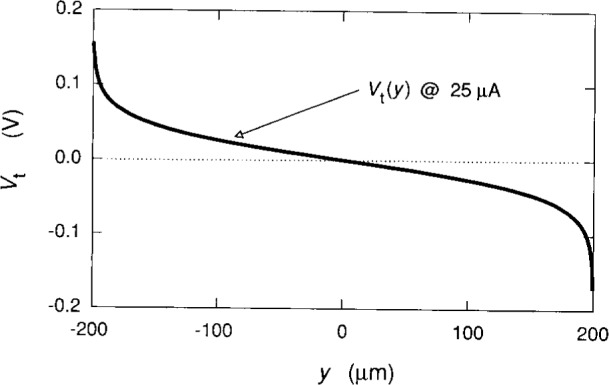
*V*_t_(*y*) at *I*_SD_ = 25 μA. This potential is in excellent agreement with the experimental measurements shown in Fig. 6 of Fontein et al. [8].

Figure 7 shows *V*_t_(*y*) at *I*_SD_ = 25 μA. The shape of this predicted potential is in excellent agreement with the experimental measurements shown in Fig. 6 of Fontein et al. [8]. It is this agreement which provides the best verification of our results. The “linear” part of the potential distribution within the sample interior, attributed in Ref. [8] to heating effects which cause *R_x_* = *V_x_*/*I*_SD_ to increase, is accounted for by our charge-redistribution potential in a sample which has minimal heating at these currents [24].

**Fig. 8:**
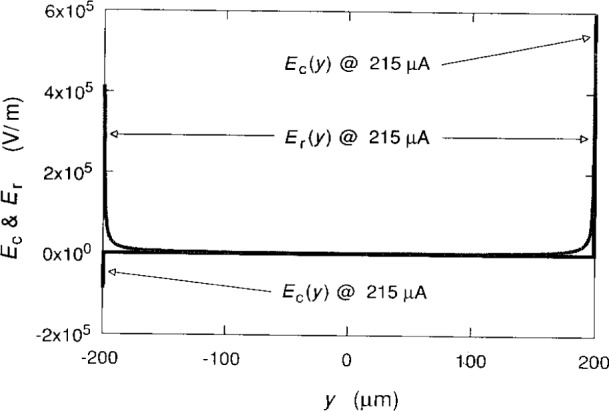
Electric fields *E*_c_(*y*) = − ∇*V*_c_(*y*) and *E*_r_(*y*) = − ∇*V*_r_(*y*) for *I*_SD_ = 215 μA.

The electric fields *E*_c_(*y*) = − **∇**_c_(*y*) and *E*_r_(*y*) = − **∇***V*_r_(*y*) are shown in Fig. 8 for *I*_SD_ = 215 μA; they were determined from Eqs. (2), (3), (18), and (20). The value of *y*_max_ = 199.599 μm is such that *E*_t_(*y*_max_) = 1.1×10^6^ V/m in equation (23). The contribution to the total electric field at *y*_max_ is slightly more for the confining potential than for the charge-redistribution potential at this current. Table 1 shows that the confining potential also provides the dominant contribution to *E*_t_(*y*_max_) at other currents.

The location, *y*_max_, of the last-filled state on the right-hand side of the sample increases with applied current *I*_SD_. We can use Eq. (A-3) and Table 1 to determine what part of this increase in *y*_max_ is due to the increase in the total electric field at *y*_max_. The percentage contributions, relative to the values of *y*_max_ and *E*_t_(*y*_max_) at *I*_SD_ = 0 μA, are 4 %, 5 %, and 3 %, for *I*_SD_ = 25 μA, 215 μA, and 225 μA, respectively. Therefore, most of the increase in *y*_max_ is due to the Lorentz force pushing the electrons closer to the side of the sample.

The current density *J*_t_(*y*) for electrons moving in the positive *x* direction is

$$J_{t}(y)=\sigma_{xy}E_{t}(y)=\frac{ie^{2}}{h}[E_{c}(y)+E_{r}(y)].$$ (26)

**Fig. 9:**
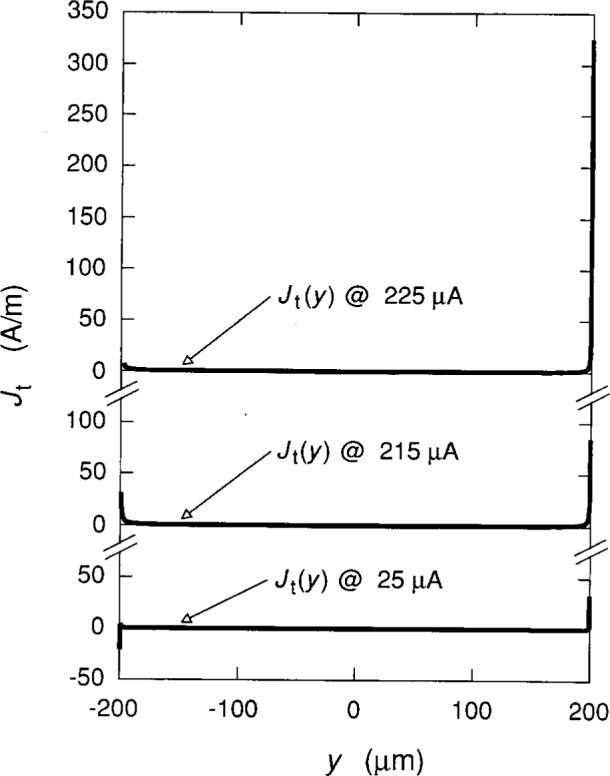
Current density *J*_t_(*y*) for *I*_SD_ = 25 μA, 215 μA, and 225 μA.

Figure 9 shows *J*_t_(*y*) for *I*_SD_ = 25 μA, 215 μA, and 225 μA. The maximum two-dimensional current density is at *y*_max_, and is 85 A/m and 325 A/m, respectively at *I*_SD_ = 215 μA and 225 μA. There is current in the negative *x* direction in the vicinity of *y*_min_ at small currents due to the dominance of the confining potential. When *I*_SD_ = 215 μA and 225 μA, however, *E*_r_(*y*_min_) > |*E*_c_(*y*_min_)| and no current flows in the −*x* direction anywhere across the sample.

The current *I* (*y*) for electrons moving in the positive *x* direction is

$$I(y)=\int_{0}^{y}J_{t}(y)dy=-\frac{V_{t}(y)}{R_{H}},$$ (27)

where

$$I_{\text{SD}}=\int_{y_{\min}}^{y_{\max}}J_{t}(y)dy=I(y_{\max})+I(y_{\min}),$$ (28)

and

$$\Delta I=I(y_{2})-I(y_{1}).$$ (29)

**Fig. 10:**
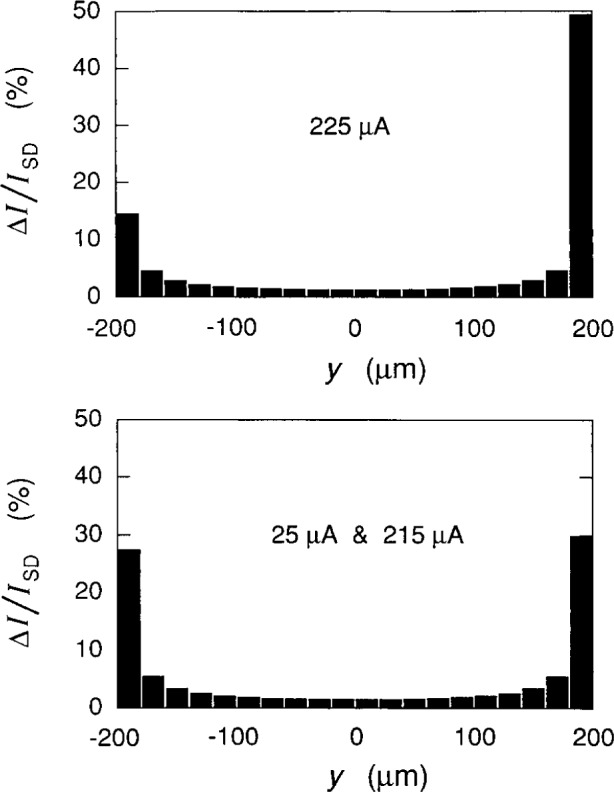
Percentage of current flowing through 20 equal segments across the sample width for *I*_SD_ = 25 μA, 215 μA, and 225 μA.

We divide the sample width into 20 equal segments in Fig. 10 and determine the percentage of current flowing through each segment for *I*_SD_ = 25 μA, 215 μA, and 225 μA. We do not show a plot for *I*_SD_ = 0 μA, but **Δ***I* would be −0.81 μA and +0.81 μA for the left-hand side and right-hand side segments, respectively, and zero for the other 18 segments because *I*_c_(rhs) = –*I*_c_(lhs) = 0.81 μA.

The current distributions in Fig. 10 are virtually identical between 25 μA and 215 μA, even though large numbers of electrons are being excited into higher Landau levels at 215 μA. The left and right side distributions are nearly symmetric. There is, however, a significant transfer of current from the left-hand side segment to the right-hand side segment at 225 μA. We saw in Sec. 5.2 that no current flows within 60, 55, and 13 magnetic lengths of the sample side for *I*_SD_ = 25 μA, 215 μA, and 225 μA, respectively. Also, 68 %, 70 %, and 51 % of the current is in the 19 segments to the left of the right-hand side segment where the edge channel current would flow for these three applied currents. The current density was negative in the left-hand side of Fig. 9 at *I*_SD_ = 25 μA because electrons were flowing in the *–x* direction at *y*_min_ −199.554 μm, but that contribution to **Δ***I* in the left-hand side segment of Fig. 10 is so small that the net current is positive.

**Fig. 11:**
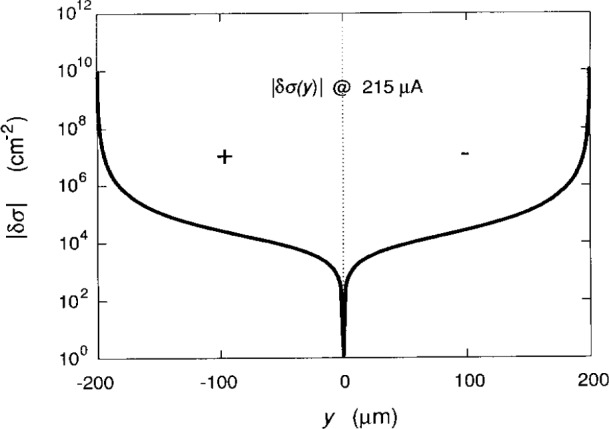
Logarithmic plot of the charge-redistribution |δ*σ* (*y*)| across the sample for *I*_SD_ = 215 μA, where δ*σ* (*y*) is the deviation from the average number density *n_s_.* The “−” region represents an excess of electrons, the “+” region a depletion of electrons.

Finally, we investigate the charge-redistribution –*e*δ*σ* (*y*) of the electrons in the 2DEG in terms of the deviation δ*σ* (*y*) in the number of electrons/cm^2^ from the average number *n*_s_ = 5.94×10^11^/cm^2^ on the *i* = 2 plateau at 12.3 T, where

$$\delta \sigma (y)=\frac{im*}{hB}\frac{d^{2}}{dy^{2}}V_{t}(y)$$ (30)

from Eq. (A-5). Figure 11 is a logarithmic plot of |δ*σ* (*y*)| versus *y* for *I*_SD_ = 215 μA. There is an excess of electrons on the +*y* side of the sample, and a depletion on the −*y* side.

An assumption made in deriving Eq. (A-5) was that the charge density varies slowly across the sample, i.e., that *ρ* (*y*) ≈ *ρ* (*y*+d*y*), or δ*σ* (*y*) *<< n*_s_. This assumption is valid here because the largest value of δ*σ* (*y*) occurs at *y*_max_, and is 2 %, 2 %, and 6 % of *n*_s_ at *I*_SD_ = 25 μA, 215 μA, and 225 μA, respectively.

One of the consequences of our approximate form of the charge-redistribution potential is that the net charge does not vanish when the charge-redistribution –*e*δ*σ* (*y*) sample width. The area under the curves in Fig. 11 is 4 % larger for the +*y* side than for the −*y* side. Therefore, there is an unaccounted excess of electrons; so this is not quite the actual shape of the charge-redistribution function. However, it is the potential and current distributions that are of primary importance to the transport properties—not the charge-redistribution. The charge was certainly conserved in the experiment of Fontein et al. [8], and yet their measured potential distributions are symmetrical. This fact demonstrates that the slight charge asymmetry does not significantly affect the potential and current distributions.

We could conserve the charge by adjusting the origin slightly to the right until the area under the curves are equal for ±*y* in Fig. 11, and then self-consistently recalculating the potentials with the new coordinates. This would greatly complicate the calculations however, and with all the approximations that have been made in this paper, and with the excellent agreement with experiment [8], it seems unnecessary. It may be a consequence of this charge nonconservation problem that the value of *y*_min_ is inside the confining potential for the case in Table 1 when *I*_SD_ = 225μA.

### 5.4 Sample-Width Dependence of the Critical Current

Balaban, Meirav, and Shtrikman [10] have found that the critical current for breakdown of the quantum Hall effect, *I*_cr_, scales logarithmically with the sample width *w* for all Landau levels. We verify this dependence by: (a) using the result in Sec. 3.3 that *I*_c_(rhs) = − *I*_c_(lhs) = 0.81 μA for the *i* = 2 plateau at 12.3 T when *I*_SD_ = 0 μA; (b) calculating the value of *y*_max_ from Eqs. (2) and (5) for each value of *w* at *I*_SD_ = 0 μA; (c) calculating the value of *G* from Eq. (17) for each value of *w*; (d) defining *I*_cr_ as the applied current *I*_SD_ sufficient to excite the lowest, *M* = 1, QUILLS transitions [11,25–28]; (e) assuming the value of *E*_c_(*y*_max_) is the same for all values of *I*_SD_ that excite *M* = 1 QUILLS transitions (The value used is *E*_c_(*y*_max_) = 5.96×10^5^ V/m, obtained from Table 1 at *I*_SD_ = 215 μA, *w* = 400 μm, and *E*_t_(*y*_max_) = 1.1×10^6^ V/m. This is equivalent to fixing the value of *I*_c_(rhs) to be 2.30 μA for each value of *I*_cr_); (f) calculating the value of *y*_max_ from Eq. (2) for each value of *w*; (g) adjusting the value of *I*_r_ so that *E*_t_(*y*_max_) = 1.1×10^6^ V/m in Eqs. (2), (17), (20), and (23); and (h) adjusting the value of *y*_min_ to give the correct Hall voltage for each current by using Eqs. (2), (15), (17), (21), and (22).

**Fig. 12:**
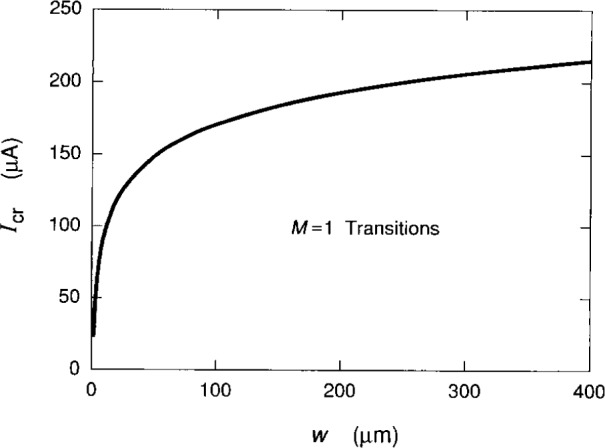
The critical current *I*_cr_ versus sample width *w*. Refer to Sec. 5.4 for details of the calculation.

The results of *I*_cr_ versus *w* are plotted in Fig. 12. The shape of the curve is identical to the experimental data of Balaban et al. [10]. The scaling is very different, however because their critical currents are about two orders of magnitude smaller than ours. We note that the experiment of Haug, von Klitzing, and Plog [29] tends to agree with the experimental curve shapes of Balaban et al. [10], but the experiment of Kawaji, Hirakawa, and Nagata [30] found a linear, rather than a logoarithmic, dependence of *I*_cr_ with *w*. Perhaps this difference is due to nonuniformities in the values of the charge-depletion width ***Δ*** along the sides of the samples, e.g., we have observed different values of *I*_cr_ along the lengths of some of our samples. If we assume that the value of *V*_m_ remains constant along a sample edge, allow ***Δ*** to vary by changing the average ionized donor density *N*_D_, and assume the ratio *E*_c_(*y*_max_)/*E*_t_(*y*_max_) remains constant, then we find that the critical current required to excite *M* = 1 QUILLS transitions with *E*_t_(*y*_max_) = 1.1×106 V/m decreases when **Δ** decreases and *N*_D_ increases, i.e., the steeper the confining potential, the smaller the critical current.

## 6. Conclusions

We have calculated potential and current distributions across the width of a GaAs/AlGaAs heterostructure sample for applied currents between 0 μA and 225 μA, using: (a) a quadratic confining potential *V*_c_(*y*) arising from charge-depletion regions along the sides of the sample; (b) parameters for that potential obtained from a localization experiment [9]; (c) a logarithmic charge-redistribution potential *V*_r_(*y*) of the 2DEG; and (d) a maximum electric field *E*_t_(*y*_max_) calculated from breakdown measurements and a QUILLS model [11]. Our predictions are in excellent agreement with experiments [8,10].

Referring to Table 1, the confining potential component *E*_c_(*y*_max_) of the electric field at *y*_max_ contributes 88 %, 54 %, and 57 % to *E*_t_(*y*_max_) at 25 μA, 215 μA, and 225 μA, respectively. The maximum current density *J*_t_(*y*_max_) is 34 A/m, 85 A/m, and 325 A/m, respectively at these three currents. A significant amount of current is distributed within the sample interior. For example, *I*_r_ is 99 %, 99 %, and 83 % of *I*_SD_, respectively at these three currents. We predict the current to be much farther from the sides of the sample than in other models, e.g., no current flows within 60, 55, and 13 magnetic lengths of the sample side for these currents. It would require a lateral resolution of about 0.1 μm, rather than the 70 μm resolution of Fontein et al. [8], to verify this result.

## Figures and Tables

**Table 1:** Values of some quantities obtained from the calculations in Sec. 5 for *I*_SD_ = 0 μA, 25μA, 215 μA, and 225 μA. The quantities common to all the calculations are *i* = 2, *B* = 12.3 T, ***κ*** = 13.1, *w* = 400 μm, ***Δ*** = 0.5 μm, *V*_m_ = 0.75 V, *a* = 3.0×10^12^ V/m^2^, *λ* = 199.500 μm, *G* = 0.147, and *l_B_* = 7.3 nm. See Secs. 2–5 for the definitions of these symbols

*I*_SD_ (μA)	*I*_c_(rhs) (μA)	*I*_c_(lhs) (μA)	*I*_r_ (μA)	*y*_max_ (μm)	*y*_min_ (μm)	*V*_c_(*y*_max_) (V)	*V*_r_(*y*_max_) (V)	*E*_c_(*y*_max_) (MV/m)	*E*_r_(*y*_max_) (MV/m)	*E*_t_(*y*_max_) (MV/m)	*δ*_max_/*l_B_*
0	0.81	−0.81	0.00	199.559	−199.559	−0.010	0.000	0.354	0.000	0.354	60.3
25	0.94	−0.68	24.74	199.564	−199.554	−0.012	−0.160	0.382	0.054	0.436	59.6
215	2.30	−0.05	212.75	199.599	−199.515	−0.030	−1.392	0.596	0.504	1.100	54.8
225	37.36	−0.00	187.64	199.901	−198.044	−0.482	−1.477	2.405	1.795	4.200	13.5

**Fig. A-1:**
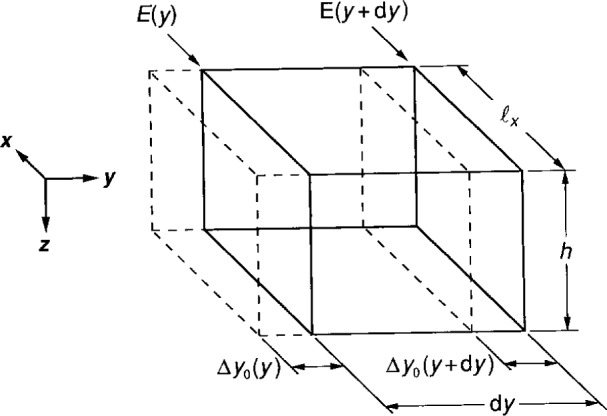
Geometry factors for the calculations in Appendix A.

## 7. Appendix A. Derivation of Eqs. (10) and (11)

We first derive Eq. (10) of Sec. 4. The Lorentz force exerted on the conducting electrons causes deviations − *e*δ*σ* (*y*′) from the average surface charge density −*en*_s_ = − *ie*^2^ *B/h* of the 2DEG at each point *y*′ across the sample width. Consider a strip of this redistributed charge of width d*y*′, located in the *x*−*y* plane at position *y*′ and pointing in the *x* direction, with a charge/length ***Λ*** (*y*′) = −*e*δ*σ* (*y*′)d*y*′. Gauss’s law is then used to obtain the electric field d*E* (*y*) at some point *y* in the *x*−*y* plane, due to one of these line charges: $\kappa \varepsilon_{0}\oint E\cdot dS=q$, and thus ***κε***_0_d*E* (*y*)2*π* |*y*′−*y* |*L_x_* = ***Λ***(*y*′)*L_x_* for a cylindrical Gaussian surface of radius |*y*′−*y*| and length *L_x_*. The potential d*V* (*y*) of this line charge is

$$\begin{array}{c} \text{dV}(y)=-\int dE(y)dy=-\frac{\Lambda (y^{\prime })}{2\pi \kappa \varepsilon_{0}}\frac{2}{w}\int_{y^{\prime }-w/2}^{y}\frac{1}{2|y^{\prime }-y|/w}dy\\ =\frac{\Lambda (y^{\prime })}{2\pi \kappa \varepsilon_{0}}\ln\frac{2|y^{\prime }-y|}{w}. \end{array}$$ (A-1)

The total potential, when summed over all the line charges, is Eq. (10)

$$V(y)=-\frac{e}{2\pi \kappa \varepsilon_{0}}\int_{-w/2}^{w/2}\delta \sigma (y^{\prime })\ln\left[\frac{2}{w}|y^{\prime }-y|\right]dy.$$ (A-2)

Now we derive Eq. (11) of Sec. 4. We found in Eq. (3) of Ref. [11] that the center of mass coordinate *y*_0_ of each state undergoing cycloidal motion in a Landau level is

$$y_{0}=\frac{E(y)}{\omega_{c}B}+\frac{\hbar k_{x}}{eB};$$ (A-3)

so the states move to the right as *E* (*y*) increases. The total charge δ*Q* transferred into the volume *l_x_h*d*y*, outlined with solid lines in Fig. A-1, is

$$\begin{array}{c} \delta Q=\delta \rho l_{x}h\;dy\;=\;l_{x}h[(\Delta y_{0}(y)\rho (y)\\ -\Delta y_{0}(y+dy)\rho (y+dy)]. \end{array}$$ (A-4)

If the volume charge density is slowly varying, then *ρ* (*y*) ≈ *ρ* (*y* + d*y*), where *ρh* ≡ −*eσ* and δ*ρh* ≡ − *e*δ*σ*. Thus, from Eqs. (A-3) and (A-4),

$$\delta \sigma (y)dy=\frac{\sigma (y)}{\omega_{c}B}[E(y)-E(y+dy)],$$

or

$$\delta \sigma (y)=-\frac{\sigma (y)}{\omega_{c}B}\frac{dE(y)}{dy}=\frac{\sigma (y)}{\omega_{c}B}\frac{d^{2}V(y)}{dy^{2}}.$$

But *σ* (*y*) = *ieB/h*, so we obtain Eq. (11)

$$\delta \sigma (y)=\frac{ie}{h\omega_{c}}\frac{d^{2}V(y)}{dy^{2}}=\frac{im*}{hB}\frac{d^{2}V(y)}{dy^{2}}.$$ (A-5)

Equation (11) considers the charge-redistribution − *e*δ*σ* (*y*) due to the second derivative of the charge-redistribution potential *V*_r_(*y*). The charge-redistribution that we calculated in Sec. 5.3 depends also on the second derivative of those regions of the *confining* potential *V*_c_(*y*) which differ from the *y*_max_ and *y*_min_ values at *I*_SD_ = 0 μA.

## 8. Appendix B. Eigenvalue Equation

We saw in Eq. (12) of Sec. 4 that

$$V_{r}(y)=-\xi \int_{-w/2}^{w/2}\frac{d^{2}}{d{y^{\prime }}^{2}}[V_{r}(y^{\prime })]\ln\left[\frac{2}{w}|y^{\prime }-y|\right]dy^{\prime },$$ (B-1)

where the characteristic length *ξ* is

$$\xi =\frac{ie^{2}}{2\pi \kappa \varepsilon_{0}h\omega_{c}}.$$ (B-2)

MacDonald, Rice, and Brinkman [19] have pointed out that Eq. (B-1) has the form of an eigenvalue equation ***Θ*** [*F*(*y*)] = *CF*(*y*), where

$$\Theta (y)=-\int_{-w/2}^{w/2}\frac{d^{2}}{d{y^{\prime }}^{2}}[\;]\ln\left[\frac{2}{w}|y^{\prime }-y|\right]dy^{\prime }$$

is an operator acting on the function *F*(*y*) = *V*_r_ (*y*′), and *C* = 1/*ξ* is a constant.

The function *F*(*y*) = *V*_r_(*y*') should have a second derivative that satisfies Eq. (B-1). Our function, from Eq. (15) of Sec. 4.1, is

$$V_{r}(y^{\prime })=-\frac{I_{r}R_{H}}{2}{\left[\ln\frac{w-\delta_{\max}}{\delta_{\max}}\right]}^{-1}\ln\left|\frac{y^{\prime }+w/2}{y^{\prime }-w/2}\right|,$$ (B-3)

where the cut-off is $\delta_{\max}=\frac{w}{2}-y_{\max}$,

$$\frac{d}{dy^{\prime }}V_{r}(y^{\prime })=\frac{I_{r}R_{H}}{2}{\left[\ln\frac{w-\delta_{\max}}{\delta_{\max}}\right]}^{-1}\frac{-w}{\left[{\left(w/2\right)}^{2}-{\left(y^{\prime }\right)}^{2}\right]},$$

and

$$\frac{d^{2}}{d{y^{\prime }}^{2}}V_{r}(y^{\prime })=\frac{I_{r}R_{H}}{2}{\left[\ln\frac{w-\delta_{\max}}{\delta_{\max}}\right]}^{-1}\frac{2wy^{\prime }}{{\left[{\left(w/2\right)}^{2}-{\left(y^{\prime }\right)}^{2}\right]}^{2}}.$$ (B-4)

We can see if the potential given by Eq. (B-3) is a valid solution to the eigenvalue expression Eq. (B-1) by substituting Eq. (B-4) into Eq. (B-1) and integrating only between the limits − *δ*_min_ to *δ*_max_ because δ*σ* (*y*′) is zero beyond these two cut-off values. Surprisingly, we obtain nearly exact solutions to the eigenvalue expression at all values of *y* when *ξ* is less than 2.0 μm and *δ*_max_ = *δ*_min_ = *ξ*. This choice of *δ*_max_ = *δ*_min_ = *ξ* for the cut-off values was used by Beenakker and van Houten [22]. Our value of *ξ* is 3.3 nm, so we would be well within this exact range if *δ*_max_ = *δ*_min_ = *ξ*. The values of *δ*_max_ do not equal *δ*_min_ however, and are much larger than the value of *ξ*. Also, we have a confining potential, *V*_c_(*y*), parts of which should be included in Eq. (B-1). It would be interesting to see how well the values of *V*_t_(*y*) obtained in Eq. (B-4) agree with the values obtained in the eigenvalue expression Eq. (B-1) when using the values of quantities obtained in Table 1.
